# The Mediating Effect of Gambling Motives between Psychiatric Symptoms, Dissociation, and Problem Gambling Severity

**DOI:** 10.1007/s10899-025-10408-9

**Published:** 2025-06-27

**Authors:** Can Zoraloğlu, Zsolt Demetrovics, Mark D. Griffiths, Andrea Czakó, Zsolt Horváth, Orsolya Király

**Affiliations:** 1 Institute of Psychology, ELTE Eötvös Loránd University, Budapest, Hungary; 2 Doctoral School of Psychology, ELTE Eötvös Loránd University, Budapest, Hungary; 3 Flinders University Institute for Mental Health and Wellbeing, College of Education, Psychology and Social Work, Flinders University, Bedford Park, SA Australia; 4 Centre of Excellence in Responsible Gaming, University of Gibraltar, Gibraltar, Gibraltar; 5 International Gaming Research Unit, Psychology Department, Nottingham Trent University, Nottingham, UK

**Keywords:** Coping, Dissociation, Escapism, Gambling Disorder, Motivation, Problem Gambling, Psychopathology

## Abstract

Although previous research has identified psychiatric symptoms and dissociation as important factors in problem gambling, the role of gambling motives in these relationships has received scant attention. According to the literature, the theoretically “riskier” motives (coping and enhancement) are associated with problem gambling. The present study examined the mediating role of gambling motives between psychiatric symptoms, dissociative experiences, and problem gambling severity. A total of 688 participants (57.8% male, M_age_ = 40.8 years, SD = 13.8) completed the Brief Symptom Inventory (BSI), Dissociative Experiences Scale-II (DES-II), Gambling Motives Questionnaire (GMQ), and Problem Gambling Severity Index (PGSI). The overall model had a good fit to the data. The results indicated that psychiatric symptoms had a significant direct effect and a significant indirect (mediating) effect on problem gambling severity via the coping motive, while dissociation only showed a significant indirect effect via the coping motive. The structural equation modeling showed that psychiatric symptoms, both directly and indirectly (via coping motives), influenced problem gambling severity, but dissociation did so indirectly only via coping motives. These results suggest that dissociation amplifies vulnerability to escape-style gambling, a concept in which an individual tries to regulate either negative mood states or psychological arousal by gambling. Awareness of dissociative tendencies and targeted interventions may help reduce gambling-related harms.

**Supplementary Information:**

The online version contains supplementary material available at 10.1007/s10899-025-10408-9.

## Introduction

Gambling research is growing, with many studies conducted to better understand its impact on psychological and psychosocial well-being. These studies have led to significant progress in understanding the clinical features of problem gambling and its treatment. However, questions on how risk factors contribute to the acquisition, development, and maintenance of problem gambling persist. According to Ferris and Wynne (2001), problem gambling is characterized by gambling behavior that results in harmful consequences for the gambler, their social circle, or the community. According to Griffiths (2016), while problem gambling may not result in negative effects across all aspects of an individual’s life, it is important to note that problem gambling and gambling disorder are not the same. They are on a continuum, with gambling disorder representing a more severe form than problem gambling. As specified in the *International Classification of Diseases*, 11th Revision (ICD-11), gambling disorder involves a recurring pattern of gambling with impaired control, prioritization over other life activities, continued gambling despite serious negative consequences in various aspects of life, and functional impairment (World Health Organization, 2019).

According to the three-dimensional model proposed by Stewart and Zack (2008), three specific motives have been identified to explain gambling behavior: *coping*, which involves using gambling as a means of reducing or avoiding negative emotions through internal, negative reinforcement; *enhancement*, which involves using gambling to increase positive emotions through internal, positive reinforcement; and *social motives*, which involve using gambling to increase social affiliation through external, positive reinforcement. Individuals who have disturbed family and personal histories, poor coping and problem-solving skills, and affective instability resulting from both biological and psychosocial deficits, may use gambling as a means to escape their emotions through dissociation or to regulate their negative mood states or physiological arousal levels (Blaszcynski & Nower, 2002). Potenza et al. (2019) suggest that these individuals may be motivated by negative reinforcement, which involves using gambling to escape emotions or negative mood states, which is consistent with the coping motive proposed by Stewart and Zack’s (2008) three-dimensional gambling motives model.

As highlighted by Raylu and Oei (2002) and Johansson et al. (2009), negative mood states play an important role in the development and maintenance of maladaptive gambling activities. According to Jacobs (1988), individuals who engage in addictive behaviors may become disrupted from their immediate surroundings and develop a distorted perception of the passage of time. This can contribute to the maintenance of addictive behaviors because individuals may become preoccupied with the pleasure and reinforcing aspects of their behavior, causing them to lose touch with the reality of their situation and become less aware of the negative consequences associated with their actions. Because gambling behavior is complex and influenced by multiple factors, dissociation has become an important factor that requires a comprehensive investigation to better understand its impact on such behavior.

In the fifth edition of the *Diagnostic and Statistical Manual of Mental Disorders* (DSM-5), dissociation is characterized by a disruption of and/or discontinuity in the normal integration of consciousness, memory, identity, emotion, perception, body representation, motor control, and behavior (American Psychiatric Association, 2013). Griffiths et al. (2006) stated that dissociative behaviors can be described as a range of experiences that exist on a continuum, which may include losing track of time, feeling a sense of detachment from oneself, experiencing temporary memory loss or blackouts, being in a trance-like state, or not being able to recall how one arrived at a specific location or what activities one engaged in, and in extreme cases, it can involve multiple personality disorders. According to Jacobs (1986), having a high level of dissociation increases the risk of developing addictive behavior. Additionally, excessive or long-term dissociation may impair cognitive and emotional functioning by making it difficult for individuals to process effectively and respond to their environment. Therefore, it is important for individuals to be aware of their own dissociative tendencies and seek appropriate support and treatment when necessary.

Various studies have reported significant associations between dissociation and disordered gambling (Cartmill et al., 2015; Craparo et al., 2015; Diskin & Hodgins, 2001; Dixon et al., 2019; Gori et al., 2016; Kofoed et al., 1997; Murch & Clark, 2019; Murch et al., 2017). Moreover, research has indicated that problem gamblers tend to use maladaptive coping styles (Gupta et al., 2004) and experience elevated levels of excitement, relaxation, and a sense of escape during gambling, as well as dissociative states more frequently than non-problem gamblers (Wood et al., 2004). Based on these findings, Wood and Griffiths (2007) suggested that individuals may turn to gambling as a way of dealing with the stresses of daily life. They may use gambling to regulate their negative mood states or physiological arousal levels, and this can lead them to seek an escape from their daily routine or life problems. Gori and Topino (2024) demonstrated that the dissociative subdimension of absorption significantly mediated the relationship between alexithymia and gambling severity, suggesting that dissociative processes may function as maladaptive coping mechanisms. Their results also indicated that individuals with a heightened external locus of control are particularly vulnerable to using gambling as an escape strategy to manage emotional dysregulation. Therefore, dissociative processes, especially those linked to escape negative emotions or negative mood states, may play a crucial role in the appeal of escape-style gambling, as highlighted by Schluter and Hodgins (2019), and further supported by earlier conceptualizations (Cartmill et al., 2015; Jacobs, 1988). Based on previous literature, it is posited that psychiatric symptoms, dissociation, problem gambling severity, and potential contributing factors, such as gambling motivations, are all associated with each other.

Psychiatric symptoms (such as anxiety, depression, and stress) and/or dissociation may increase the likelihood of individuals using maladaptive coping mechanisms, and gambling can be one such mechanism, providing temporary relief. According to the Self-Medication Hypothesis, individuals engage in behaviors such as gambling to regulate negative affective states (Khantzian, 1997). Rogier et al. (2021) suggested that individuals at risk of developing gambling disorder tend to use dysfunctional emotion regulation strategies that increase their emotional arousal, which does not resolve emotional issues but instead amplifies the individual’s emotional distress, making the need for emotional regulation even greater. Consequently, this increased distress makes the individual more likely to engage in escape-based strategies, such as gambling, to escape the emotional discomfort. Therefore, individuals who use gambling as a coping strategy may be more likely to develop problematic gambling behaviors. This is because they might use gambling more frequently and intensively as a means of coping, leading to an increase in gambling-related problems. Gambling motives, particularly coping motives may link the psychological need to manage stress and negative emotions (arising from psychiatric symptoms) with the behavioral outcome of problem gambling. Essentially, coping motives may be crucial in explaining the pathway from psychiatric symptoms to problem gambling by clarifying how and why individuals with psychiatric symptoms might turn to gambling, rather than relying on other motives and/or engaging in different maladaptive behaviors, thereby leading to problem gambling. Although prior research has examined associations between psychiatric symptoms, dissociation, and problem gambling, few studies have explicitly explored the mechanisms that explain these relationships. Most existing studies focus on direct relationships without investigating the underlying processes driving these associations.

The present study sought to address this gap by examining gambling motives, particularly coping motives, as a mediating factor in the relationship between psychiatric symptoms, dissociation, and problem gambling severity. Escapism, often conceptualized as a form of avoidance coping, refers to using gambling as a means to disengage from reality, suppress negative emotions, or distract from distressing thoughts. Given that dissociation itself involves a psychological detachment from one’s surroundings, escapism may serve as a critical link between dissociative tendencies and problematic gambling behaviors. The present study contributes to the literature by integrating theoretical perspectives from gambling motives research and dissociation literature within a mediation model. By identifying coping motives as a key mechanism, the study provides a more nuanced understanding of why individuals with psychiatric symptoms or dissociative tendencies develop problem gambling. This perspective has significant implications for intervention strategies, because it highlights the importance of addressing maladaptive self-regulation mechanisms rather than solely targeting gambling behaviors. Consequently, the present study aims to expand current knowledge and offer insights that can inform more effective prevention and treatment approaches for problem gambling. Based on the literature, it was hypothesized that coping motives would function as a mediator within the proposed model.

## Methods

### Participants and Procedure

Participants comprised adult gamblers living in Budapest (Hungary). They were recruited in lottery shops, casinos, and gaming arcades. After a brief verbal description of the study, individuals who agreed to participate in the study completed a consent form and either completed the questionnaire in a pen-and-paper format at the venue (18% of the participants) or provided their email addresses to participate online. Unique passwords were sent to them via email, with which they could log in to the study platform and complete the survey online, which took approximately 20–30 min. The data collection lasted for three months. The research protocol was reviewed and approved by the Research Ethics Committee at ELTE Eötvös Loránd University. Participation in the study was voluntary and anonymous (email addresses were kept separately from the survey responses). The study did not collect or store any personal information from participants and did not offer any incentives for participation.

### Measures

#### Sociodemographic Variables

Major sociodemographic data, such as age, gender, marital status, education, and occupation, were collected at the beginning of the survey.

#### Gambling-Related Variables

Data were collected regarding different types of gambling and their frequency (i.e., playing cards with money; betting on animal races (e.g., horses, greyhounds, etc.); sports betting; dice game gambling; land-based casino gambling; number pools, lottery or other draw-based games; scratch-cards; stock market trading; slot machine gambling; online casino gambling; betting money on the outcome of skill-based games such as billiards, bowling, or golf; and ‘other’ forms of gambling, similar to other studies (i.e., Gyollai et al., 2013). The response options were given for each gambling type (“No” = 0; “Yes” = 1) and the frequency of each of them. The question asked whether the participant played the specific type of gambling weekly or more frequently and the response options were the following: “I never played weekly or more frequently” = 0; “More than a year ago” = 1; “Over the past year” = 2; “Over the past month” = 3.

#### Psychiatric Symptoms

The 53-item Brief Symptom Inventory (BSI; Derogatis, 1983; Hungarian version: Urbán et al., 2014) was used to assess psychiatric symptoms. Items (e.g., *“During the past seven days*, *how much were you distressed by feeling easily annoyed or irritated”*) are rated using a five-point Likert scale from 0 (*not at all*) to 4 (*extremely*) to assess nine dimensions of psychological symptoms (somatization, obsession-compulsive, interpersonal sensitivity, depression, anxiety, hostility, phobia, paranoia, and psychoticism). The mean score of all 53 items (the Global Severity Index [GSI]), was used to indicate the intensity level of psychiatric symptoms. The BSI has been shown to have good reliability and validity in various samples (Derogatis, 1975, 1993). In the present study, the internal consistency was excellent (*α* = 0.94).

#### Dissociative Experiences

The 28-item Dissociative Experiences Scale-II (DES-II; Carlson & Putnam, 1993) was used to assess the frequency of dissociative experiences. Items (e.g., *“Some people have the experience of finding themselves in a place and have no idea how they got there”*) are rated using an 11-point Likert scale (from 0 to 100% of the time) to assess how often individuals experience dissociative symptoms as a percentage of the time. The scale has a total score (DES-II total score) that indicates an individual’s general level of dissociation, as well scores for three subtypes of dissociation (dissociative amnesia, absorption, and depersonalization-derealization). Higher scores on the DES-II indicate a higher level of dissociation. Only the DES-II total score was used in the present study, and the internal consistency was very good (*α* = 0.88).

#### Gambling Motives

The 15-item Gambling Motives Questionnaire (GMQ; Stewart & Zack, 2008) was used to assess three dimensions of gambling motivation: enhancement (e.g., *“because it’s exciting”*), social (e.g., *“to be sociable”*), coping (e.g., *“because it helps when you are feeling nervous or depressed”*). Items are rated on a four-point Likert scale from 1 (*almost never/never*) to 4 (*almost always*), with higher scores indicating a higher frequency of the respective motivational dimension. Internal consistency was very good for enhancement (*α* = 0.88), good for coping (*α* = 0.73), and poor for social motives (*α* = 0.56).

#### Problem Gambling Severity

The 9-item Problem Gambling Severity Index (PGSI; Ferris & Wynne, 2001; Gyollai et al., 2013) was used to assess problem gambling. Items (e.g., *“Have you bet more than you could really afford to lose?”*) are rated on a 4-point Likert scale from 0 (*never*) to 3 (*almost always*). The scores of the nine items are summed to produce a score for problem gambling severity and higher scores on the PGSI indicate a greater risk for problem gambling. Based on the conventional scoring system, total scores are categorized as follows: 0 = non-problem gambler, 1–2 = low-risk gambler, 3*–*7 = moderate-risk gambler, and 8 or more = problem gambler. In the present study, the internal consistency of the PGSI was good (*α* = 0.76).

### Statistical Analyses

Structural equation modeling (SEM) was used to test the proposed mediation model using structural regression analysis. The scales used in the study were not normally distributed. However, due to the use of bootstrapping procedures, the maximum likelihood (ML) estimator was applied, as recommended for such analyses in MPlus (Muthen & Muthen, 1998–2017). To assess the significance of indirect (mediated) effects, bias-corrected bootstrapping with 10,000 resamples was conducted. This non-parametric resampling method provides robust confidence intervals for indirect effects, especially in cases where the sampling distribution may be skewed or non-normal. To evaluate the fit of the overall models, the chi-square goodness-of-fit statistic (with a *p* value < 0.05), the comparative fit index (CFI), the Tucker-Lewis fit index or non-normed fit index (TLI or NNFI), root mean square error approximation (RMSEA) and its 90% confidence interval (90% CI), and the standardized root mean square residuals (SRMR) were used. A good fit is indicated by values greater than 0.9 for CFI and TLI, and values less than 0.08 for RMSEA and SRMR (Browne & Cudek, 1993; Kline, 2023). Descriptive analyses were conducted using SPSS 28.0 (IBM Corp, 2021) and all SEM analyses were performed using MPlus 8.0 software (Muthen & Muthen, 1998–2017).

## Results

### Descriptive Statistics

The mean age of the sample (*N* = 688) was 40.8 years (SD = 13.8; range 20–86 years), and the percentage of male participants was higher than female participants (57.8%). Among gambling types, the most common ones were number pools, lottery or other draw-based games (91.0%), buying scratch cards (70.2%), and sports betting (59.7%). For other gambling types and their frequencies see Table 1.

**Table 1:** Sample characteristics (*N* = 688)

Variable		Frequency	Percentage (%)
Age (in years)	M (SD) = 40.8 (13.8)		
Gender			
	Male	398	57.8
	Female	290	42.2
Marital Status			
	Unmarried, single	175	25.4
	In a relationship, not married	206	29.9
	Married	225	32.7
	Divorced	69	10
	Widow	13	1.9
Level of Education			
	Elementary school	15	2.2
	Vocational school	60	8.7
	High school	327	47.5
	College or university degree	286	41.6
Currently Studying			
	Yes	216	31.4
	No	473	68.6
Working Status			
	Yes	507	73.7
	No	181	26.3
Problem Gambling Severity	M (SD) = 0.87 (1.7)		
	Non-problem gambler	459	66.7
	Low-risk gambler	145	21.1
	Moderate-risk gambler	72	10.5
	Problem gambler	12	1.7
Gambling Type Preferences (life-time occurrence in the sample)			
	Number pools, lottery or other draw-based games	626	91.0
	Scratch cards	483	70.2
	Sports betting	410	59.6
	Playing cards with money	226	32.8
	Slot machine	192	27.9
	Land-based casino	140	20.3
	Playing skilled-based games such as billiards, bowling or golf with betting money on the outcome	111	16.2
	Betting on horse racing, dog running or other animals	60	8.7
	Stock exchange	58	8.4
	Online casino	44	6.4
	Dice game	19	2.8
	Other	64	9.4
Occurrence of Frequent (weekly or more) Gambling in the Sample			
	Number pools, lottery or other draw-based games	147	21.4
	Scratch cards	201	29.2
	Sports betting	121	17.6
	Playing cards with money	83	12.1
	Slot machine	86	12.5
	Land-based casino	66	9.6
	Playing skilled-based games such as billiards, bowling or golf with betting money on the outcome	41	6.0
	Betting on horse racing, dog running or other animals	29	4.2
	Stock exchange	24	3.5
	Online casino	12	1.7
	Dice game	5	0.7
	Other	16	2.3
Psychiatric Symptoms	M (SD) = 0.38 (0.38)		
Dissociative Experiences	M (SD) = 0.58 (0.61)		

The majority of the participants were in a relationship (62.6%), had a job (73.7%), were not currently studying as a student (68.6%), and had a high school degree as their highest level of education (47.5%). Based on their PGSI scores, the majority of the sample were non-problem gamblers (66.7%), with the remainder being low-risk gamblers (21.1%); moderate-risk gamblers (10.5%), and problem gamblers (1.7%). Information regarding further sample characteristics is shown in Table 1.

### Mediation Analyses

The study’s hypothesis was that psychiatric symptoms and dissociation would have both a direct and indirect effect on problem gambling severity via the mediating effect of the three online gambling motives. Psychiatric symptoms, dissociative experiences, and problem gambling severity were introduced in the model as continuous observed variables. Gambling motives were introduced in the model as continuous latent variables. The proposed mediation model was tested with SEM. The overall model had a good fit to the data (χ^2^_667_ = 845.4, *p* <.001; CFI = 1.00; TLI = 1.00; RMSEA = 0.00, 90% CI 0.00–0.00; Cfit > 0.90; SRMR = 0.00). Psychiatric symptoms demonstrated both direct and indirect effects on problem gambling severity, whereas dissociative experiences demonstrated only an indirect effect on problem gambling severity. The findings were consistent with the study’s hypothesis. The coping motive emerged as a significant mediator, partially accounting for the relationship between psychiatric symptoms, dissociation, and problem gambling severity. Bivariate correlations between the variables are presented in Table 2.

**Table 2:** Bivariate correlations between the study variables

	1	2	3	4	5	6	7	8
1. Gender	-							
2. Age	0.12**	-						
3. Dissociative experiences	0.01	− 0.13**	-					
4. Psychiatric symptoms	0.05	− 0.07	0.47**	-				
5. Coping motives	− 0.13**	0.07	0.23**	0.29**	-			
6. Enhancement motives	− 0.24**	− 0.13**	0.22**	0.17**	0.62**	-		
7. Social motives	− 0.18**	− 0.09*	0.22**	0.16**	0.65**	0.62**	-	
8. Problem gambling severity	− 0.24**	− 0.07	0.28**	0.38**	0.56**	0.46**	0.37**	-

*p <.05 **p <.01

**Fig. 1:**
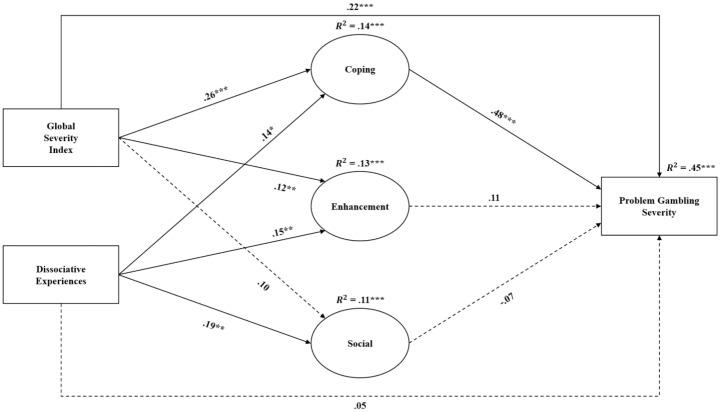
The overall mediation model with standardized path coefficients and the explained variance of the endogenous variables (R^2^) (*N* = 688) *Notes*. ML estimation with 10,000 bias-corrected bootstrap resamples was applied. All three mediator variables are latent variables. Also, for clarity, the covariances between the errors of all mediator variables have not been depicted in the figure. Simple arrows: significant path coefficients, dotted arrows: non-significant path coefficients. *p <.05; **p <.01; ***p <.001

Table 3; Fig. 1 show the results of the mediation model. The results indicated that psychiatric symptoms had a significant direct effect on problem gambling severity (*β* = 0.22, *p* <.001) as well as on the coping motive (*β* = 0.26, *p* <.001) and enhancement motive (*β* = 0.12, *p* <.01). Furthermore, dissociative experiences had a significant direct effect on coping motive (*β* = 0.14, *p* <.05), enhancement motive (*β* = 0.15, *p* <.01), and social motive (*β* = 0.19, *p* <.01). There was no significant direct effect of dissociative experiences on problem gambling severity.

**Table 3:** Predictive effects in the mediation model

Predictor variables					Outcome variables			
	Coping		Enhancement		Social		Problem Gambling Severity	
	β (S.E.)	CI (95%)	β (S.E.)	CI (95%)	β (S.E.)	CI (95%)	β (S.E.)	CI (95%)
Gender	− 0.16 (0.03)***	− 0.21– − 0.11	− 0.24 (0.03)***	− 0.30– − 0.18	− 0.18 (0.03)***	− 0.23– − 0.13	− 0.17 (0.03)***	− 0.21– − 0.13
Age	0.09 (0.05)	0.02– 0.17	− 0.09 (0.04)*	− 0.16– − 0.03	− 0.06 (0.05)	− 0.14– 0.02	− 0.03 (0.03)	− 0.08– 0.01
Dissociative experiences	0.14 (0.06)*	0.04– 0.23	0.15 (0.05)**	0.08– 0.22	0.19 (0.06)**	0.10– 0.29	0.05 (0.05)	− 0.02– 0.13
Psychiatric symptoms	0.26 (0.06)***	0.16– 0.36	0.12 (0.04)**	0.05– 0.20	0.10 (0.06)	0.01– 0.19	0.22 (0.05)***	0.13– 0.31
Coping motives	-		-		-		0.48 (0.07)***	0.36– 0.59
Enhancement motives	-		-		-		0.11 (0.06)	0.02– 0.21
Social motives	-		-		-		− 0.07 (0.06)	− 0.17– 0.04
Explained variance (R^2^)	14%		13%		11%		45%	
Correlations between the variables	Coping-Enhancement: *r* =.57*** Coping-Social: *r* =.64*** Enhancement-Social: *r* =.57***							

*Notes*: ML estimation with bias-corrected bootstrapping (10,000 resamples) was used to address non-normality and assess indirect effects. *β*: Beta coefficient, the strength and direction of the relationship between variables. *S.E.*: standard error, estimate of the variability or precision of the beta coefficient. *r* (correlation coefficient): Strength and direction of the linear relationship between two variables. CI 95% = 95% Confidence Interval * *p* <.05; ** *p* <.01; *** *p* <.001

In relation to the association between motives and problem gambling, only the coping motive had a considerable effect size (*β* = 0.48, *p* <.001). In relation to the indirect effects between psychiatric symptoms, dissociative experiences, and problem gambling severity, two paths were statistically significant: (i) psychiatric symptoms → coping → problem gambling severity (*β* = 0.12, *p* <.01) and (ii) dissociative experiences → coping → problem gambling severity (*β* = 0.07, *p* <.05). Effect sizes for the total, direct, total indirect, and specific indirect paths are presented in Table 4. The proportion of the mediated effect in the total effect was 38% for the psychiatric symptom pathways, and 58% for dissociative experiences. Therefore, both higher levels of psychiatric symptoms and dissociative experiences were associated with higher coping motives that were associated with a higher level of problem gambling. All other indirect pathways were non-significant (*p* >.05). The full model explained 45% of the total variance of problem gambling severity.

**Table 4:** Total, direct, total indirect, and specific indirect effects

	Problem Gambling Severity β (S.E.)			
	Psychiatric Symptoms → Problem Gambling Severity		Dissociative Experiences → Problem Gambling Severity	
	β (S.E.)	CI (95%)	β (S.E.)	CI (95%)
Total	0.35 (0.06)***	0.25– 0.44	0.12 (0.06)*	0.02– 0.22
Direct	0.22 (0.05)***	0.13– 0.31	0.05 (0.05)	− 0.02– 0.13
Total indirect	0.13 (0.04)**	0.07– 0.20	0.07 (0.03)*	0.03– 0.12
Specific indirect				
via coping motives	0.12 (0.04)**	0.07– 0.20	0.07 (0.03)*	0.02– 0.11
via enhancement motives	0.01 (0.01)	0.00– 0.03	0.02 (0.01)	0.00– 0.04
via social motives	− 0.01 (0.01)	− 0.03– 0.00	− 0.01 (0.01)	− 0.04– 0.01

*Notes*: ML estimation with bias-corrected bootstrapping (10,000 resamples) was used to address non-normality and assess indirect effects. *β*: Beta coefficient, the strength and direction of the relationship between variables. *S.E.*: standard error, estimate of the variability or precision of the beta coefficient. *p <.05; **p <.01; ***p <.001

Gender was a control variable in the model and it had considerable effect sizes. Given the significant role of gender, follow-up analyses were conducted stratified by gender (Table S1, Table S2, Table S3, Table S4, Figure S1, Figure S2 in the Supplementary Materials). Results showed that the predictive pathways were substantially stronger among males compared to females. Among males, psychiatric symptoms predicted problem gambling severity both directly and indirectly via coping motives, while dissociative experiences had a weaker indirect effect. In contrast, for females, most predictors had small and non-significant effects. Specifically, while dissociative experiences predicted social motives, this did not translate into significant mediation paths. The variance explained in problem gambling severity was slightly higher for males (47%) than for females (40%).

## Discussion

The present study examined the mediating role of gambling motives between psychiatric symptoms and dissociation with problem gambling severity. The findings partially supported the hypotheses because psychiatric symptoms were both directly and indirectly (via the coping motive) associated with problem gambling, while dissociative experiences showed only indirect effects through this motive. The mediating effect of the motives accounted for 38% of the total effect of the psychiatric symptoms for problem gambling severity, while the mediating effect of the motives accounted for 58% of the total effect of dissociation for problem gambling severity. In addition, the model was tested separately for males and females. According to the findings, predictive relationships were notably more pronounced among males than among females. For males, psychiatric symptoms were significant predictors of problem gambling severity, both through direct effects and indirectly through coping motives, whereas dissociative experiences showed a weaker indirect effect. In comparison, most predictors had only minimal and statistically non-significant effects among females. These gendered patterns suggest that the mechanisms underlying problem gambling may operate differently across genders (Wong et al., 2013), warranting further exploration in future studies.

To the best of the authors’ knowledge, the present study is the first in which an SEM framework has been used to investigate the associations between psychiatric symptoms, dissociation, gambling motives, and problem gambling severity. The results regarding the associations between motives and problem gambling severity are consistent with previous findings in the literature. Coping motives have been strongly associated with problem gambling in previous studies (Dechant, 2014; Milosevic & Ledgerwood, 2010). Additionally, it was found that escape (coping) motives were the best predictor of problem gambling, followed by excitement (enhancement) motives (Flack & Morris, 2015). Stewart and Zack (2008) found that the theoretically “riskier” motives (coping and enhancement) predicted the severity of problem gambling. However, when gambling behavior levels were controlled for, only coping motives remained a significant predictor of problem gambling severity.

The mean prevalence rates of mood disorders and anxiety disorders among individuals with disordered gambling were 37.9% and 37.4%, respectively, according to a meta-analysis conducted by Lorains et al. (2011). These results suggest that individuals with gambling disorder are far more likely to experience mood and anxiety disorders compared to the general population. Additionally, this association may also be bi-directional, meaning that anxiety and mood problems may increase vulnerability to disordered gambling. Research indicates that individuals with psychopathology due to traumatic experiences might engage in gambling as a way to escape from negative emotions or negative mood states, rather than seeking social benefits (Ledgerwood & Milosevic, 2015; Ledgerwood & Petry, 2006).

The findings of the present study were consistent with previous research on the relationship between motives and psychiatric symptoms. Psychiatric symptoms were moderately correlated with dissociation, and findings showed that coping motives had the strongest relationship with psychiatric symptoms. On the other hand, the relationship between dissociation and the three gambling motives showed fairly equal strength, with all the correlations being statistically significant. The present study showed that coping motives mediated between both psychiatric symptoms and dissociation, and problem gambling severity. Using gambling to escape negative emotions or negative mood states appears to be a motivation that is associated with both psychiatric symptoms and dissociation, and also predicts the severity of problem gambling. These findings support the self-medication theory (Khantzian, 1985) within the domain of problem gambling, which originally proposed that individuals may engage in substance use as a mechanism for coping with psychiatric symptoms.

Similarly, Jacobs suggested (1988) that individuals with addictions who share common dissociative-like experiences may engage in activities as a way to self-treat and escape negative feelings such as stress or unhappiness. This altered state of awareness also has the potential to lower awareness of negative mood states or ameliorate life problems, making them less overwhelming and easier to manage. Consequently, dissociation may facilitate a temporary escape from the stressors and challenges of daily life by providing individuals with a coping mechanism. However, as noted by Király et al. (2015), because escaping real-life problems only provides temporary relief from perceived stress, and maintains or even increases the severity of the original problem, in this context, it appears to be a maladaptive coping mechanism in the long-term.

As expected, enhancement and social motives did not mediate either between psychiatric symptoms and problem gambling severity or between dissociation and problem gambling severity. This finding strengthens the hypothesis regarding the role of coping motives in the association between psychiatric symptoms, dissociation, and problem gambling severity. According to previous findings in the literature, coping was the only significant predictor of problem gambling severity among gambling motives, while other motives indirectly contributed to the prediction of severity through their associations with gambling frequency (Schellenberg et al., 2016). Therefore, individuals who are motivated by negative reinforcement (using gambling as an escape from negative emotions or negative mood states) would be at a particularly high risk of experiencing gambling problems, while positive reinforcement motives such as enhancement or social factors may be relatively less important in the development and maintenance of gambling problems.

Although the relatively large sample size offered specific advantages, there are a number of limitations that should be noted concerning the present study. Any claims of generalizability to other populations are limited because the study only included a self-selected sample of Hungarian participants which was slightly gender-biased towards males. Future studies should replicate the present findings using a broader and more varied sample that includes participants that are gender-balanced from different nationalities, particularly those with more severe gambling problems. It is important to acknowledge that, due to the cross-sectional design of the study, establishing a causal interpretation of the observed relationships was not possible, and the directionality of these relationships could not be established. Therefore, using longitudinal or experimental approaches may provide a more comprehensive understanding of the topic and allow for the establishment of causal relationships regarding the proposed model. Additionally, it is important to consider and empirically examine alternative models too that act in the opposite direction, in which problem gambling leads to psychiatric symptoms and/or dissociation. Future research should explore alternative hypotheses suggesting that problem gambling may contribute to the development and maintenance of psychiatric symptoms and/or dissociation.

## Conclusion

The present study adds to the ever-growing complexity in the field of behavioral addiction by investigating the underlying psychological mechanisms of problem gambling in an attempt to explain the relationships between psychiatric symptoms, dissociation, gambling motives, and problem gambling severity. The proposed mediation models suggested that both psychiatric symptoms and dissociation were indirectly (via coping motive) associated with problem gambling. Moreover, psychiatric symptoms were also directly associated with problem gambling severity, while no direct effects of dissociative experiences were found on problem gambling severity. More specifically, coping played an important role in developing and maintaining problem gambling. These findings have direct clinical and public health implications. Given that maladaptive coping and/or escapism are central pathways linking psychiatric symptoms and dissociative tendencies to problem gambling, interventions should be more focused on unhealthy coping strategies, especially among individuals with psychiatric symptoms or tendencies towards dissociation, and/or strategies for improving self-regulation mechanisms. Helping such individuals to develop healthier coping mechanisms could lower their chances of developing problem gambling and improve their general mental well-being. Furthermore, screening for dissociative tendencies among high-risk populations, such as individuals experiencing depression, anxiety, or trauma-related disorders, could facilitate early identification and intervention, potentially preventing the escalation of gambling-related problems. At a broader level, these results highlight the importance of integrating mental health support into gambling prevention programs. Public awareness campaigns and policy initiatives should emphasize the psychological underpinnings of problem gambling, promoting alternative coping strategies and mental well-being resources. Additionally, gambling platforms could implement responsible gambling measures, such as personalized feedback tools and self-exclusion options, tailored to individuals exhibiting high levels of escapism and emotion-driven gambling.

Problem gambling is of growing concern to researchers and clinicians because of the serious negative consequences that this behavior has on the lives of individuals, their families, and the broader community. Studies with longitudinal designs are needed to understand the temporal dynamics between these variables and to evaluate the efficacy of interventions targeting gambling as a coping behavior. Moreover, understanding the relationships between other contributing factors such as personality characteristics and/or cultural influences is important. These variables should be considered in future research because they may also contribute to the development of more effective prevention and intervention strategies.

## Electronic Supplementary Material

Below is the link to the electronic supplementary material.

Supplementary Material 1

## Data Availability

Data supporting the findings of this study are available from the corresponding author upon request.
