# Supplementary material for: The Mediating Effect of Gambling Motives between Psychiatric Symptoms, Dissociation, and Problem Gambling Severity
Source: J Gambl Stud. 2025 Jun 27;41(3):1229–45. doi: 10.1007/s10899-025-10408-9 (PMC12361296; doi:10.1007/s10899-025-10408-9)
Supplement: Supplementary file 1 — Supplementary Material 1 [file 10899_2025_10408_MOESM1_ESM.docx]

**Table S1**

*Predictive effects in the mediation model (for males)*

| Predictor variables | Outcome variables | | | | | | | |
| --- | --- | --- | --- | --- | --- | --- | --- | --- |
|  | Coping | | Enhancement | | Social | | Problem Gambling Severity | |
|  | *β (S.E.)* | CI (95%) | *β (S.E.)* | CI (95%) | *β (S.E.)* | CI (95%) | *β (S.E.)* | CI (95%) |
| Age | .06 (.06) | -.05 – .16 | -.09 (.05) | -.17 – .00 | -.09 (.07) | -.19 – .02 | -.06 (.04) | -.12 – .01 |
| Dissociative experiences | .14 (.08) | .00 – .26 | .14 (.06)* | .04 – .22 | .15 (.08)* | .03 – .28 | .06 (.06) | -.04 – .16 |
| Psychiatric symptoms | .34 (.08)*** | .20 – .48 | .17 (.06)** | .07 – .27 | .19 (.08)* | .06 – .33 | .30 (.07)*** | .19 – .43 |
| Coping motives | - | - | - | - | - | - | .44 (.09)*** | .29 – .59 |
| Enhancement motives | - | - | - | - | - | - | .14 (.06)* | .04 – .25 |
| Social motives | - | - | - | - | - | - | -.10 (.07) | -.22 – .03 |
| Explained variance (R^2^) | 18% |  | 6% |  | 7% |  | 40% |  |
| Correlations between the variables | Coping-Enhancement: *r* = .57*** Coping-Social: *r* = .64*** Enhancement-Social: *r* = .57*** | | | | | | | |

*Notes:* ML estimation with bias-corrected bootstrapping (10,000 resamples) was used to address non-normality and assess indirect effects. *β*: Beta coefficient, the strength and direction of the relationship between variables. *S.E.*: Standard Error, estimate of the variability or precision of the beta coefficient. *r* (Correlation Coefficient): Strength and direction of the linear relationship between two variables. CI 95% = 95% Confidence Interval * *p* <.05; ** *p* <.01; *** *p* <.001.

**Table S2**

*Predictive effects in the mediation model (for females)*

| Predictor variables | Outcome variables | | | | | | | |
| --- | --- | --- | --- | --- | --- | --- | --- | --- |
|  | Coping | | Enhancement | | Social | | Problem Gambling Severity | |
|  | *β (S.E.)* | CI (95%) | *β (S.E.)* | CI (95%) | *β (S.E.)* | CI (95%) | *β (S.E.)* | CI (95%) |
| Age | .15 (.06)* | .04 – .25 | -.11 (.06) | -.20 – .00 | -.03 (.06) | -.13 – .08 | .02 (.05) | -.07 – .11 |
| Dissociative experiences | .12 (.10) | -.04 – .30 | .17 (.09) | .03 – .33 | .28 (.10)** | .13 – .45 | .01 (.08) | -.10 – .15 |
| Psychiatric symptoms | .12 (.10) | -.03 – .29 | .08 (.08) | -.04 – .22 | .10 (.07) | -.21 – .00 | .06 (.06) | -.03 – .19 |
| Coping motives | - | - | - |  | - | - | .54 (.11)*** | .35 – .69 |
| Enhancement motives | - | - | - |  | - | - | .19 (.09)* | .05 – .34 |
| Social motives | - | - | - |  | - | - | -.10 (.10) | -.25 – .07 |
| Explained variance (R^2^) | 6% |  | 6% |  | 7% |  | 40% |  |
| Correlations between the variables | Coping-Enhancement: *r* = .57*** Coping-Social: *r* = .64*** Enhancement-Social: *r* = .57*** | | | | | | | |

*Notes:* ML estimation with bias-corrected bootstrapping (10,000 resamples) was used to address non-normality and assess indirect effects. *β*: Beta coefficient, the strength and direction of the relationship between variables. *S.E.*: Standard Error, estimate of the variability or precision of the beta coefficient. *r* (Correlation Coefficient): Strength and direction of the linear relationship between two variables. CI 95% = 95% Confidence Interval * *p* <.05; ** *p* <.01; *** *p* <.001.

**Table S3**

*Total, direct, total indirect and specific indirect effects (for males)*

|  | | Problem Gambling Severity *β (S.E.)* | | | |
| --- | --- | --- | --- | --- | --- |
|  | | Psychiatric Symptoms → Problem Gambling Severity | | Dissociative Experiences → Problem Gambling Severity | |
|  | | *β (S.E.)* | CI (95%) | *β (S.E.)* | CI (95%) |
| Total | | .46 (.07)*** | .34 – .58 | .12 (.08) | -.01 – .25 |
| Direct | | .30 (.07)*** | .19 – .43 | .06 (.06) | -.04 – .16 |
| Total Indirect | | .15 (.05)** | .08 – .26 | .06 (.03)* | .01 – .12 |
| Specific Indirect | |  |  |  |  |
|  | via Coping motives | .15 (.06)** | .01 – .05 | .06 (.03) | .01 – .12 |
|  | via Enhancement motives | .02 (.01) | .07 – .26 | .02 (.01) | .00 – .05 |
|  | via Social motives | -.02 (.02) | -.06 – .00 | -.02 (.02) | -.05 – .00 |

*Notes:* ML estimation with bias-corrected bootstrapping (10,000 resamples) was used to address non-normality and assess indirect effects. *β*: Beta coefficient, the strength and direction of the relationship between variables. *S.E.*: Standard error, estimate of the variability or precision of the beta coefficient. **p*<.05; ***p*<.01; ****p*<.001.

**Table S4**

*Total, direct, total indirect and specific indirect effects (for females)*

|  | | Problem Gambling Severity *β (S.E.)* | | | |
| --- | --- | --- | --- | --- | --- |
|  | | Psychiatric Symptoms → Problem Gambling Severity | | Dissociative Experiences → Problem Gambling Severity | |
|  | | *β (S.E.)* | CI (95%) | *β (S.E.)* | CI (95%) |
| Total | | .15 (.11) | .01 – .36 | .08 (.10) | -.06 – .26 |
| Direct | | .06 (.06) | -.03 – .19 | .01 (.08) | -.10 – .15 |
| Total Indirect | | .09 (.07) | -.00 – .23 | .07 (.07) | -.04 – .20 |
| Specific Indirect | |  |  |  |  |
|  | via Coping motives | .07 (.06) | -.01 – .18 | .07 (.06) | -.01 – .17 |
|  | via Enhancement motives | .02 (.02) | -.00 – .06 | .03 (.03) | .00 – .09 |
|  | via Social motives | .01 (.01) | -.00 – .05 | -.03 (.03) | -.09 – .01 |

*Notes:* ML estimation with bias-corrected bootstrapping (10,000 resamples) was used to address non-normality and assess indirect effects. *β*: Beta coefficient, the strength and direction of the relationship between variables. *S.E.*: Standard error, estimate of the variability or precision of the beta coefficient. **p*<.05; ***p*<.01; ****p*<.001.

**Figure S1**

*
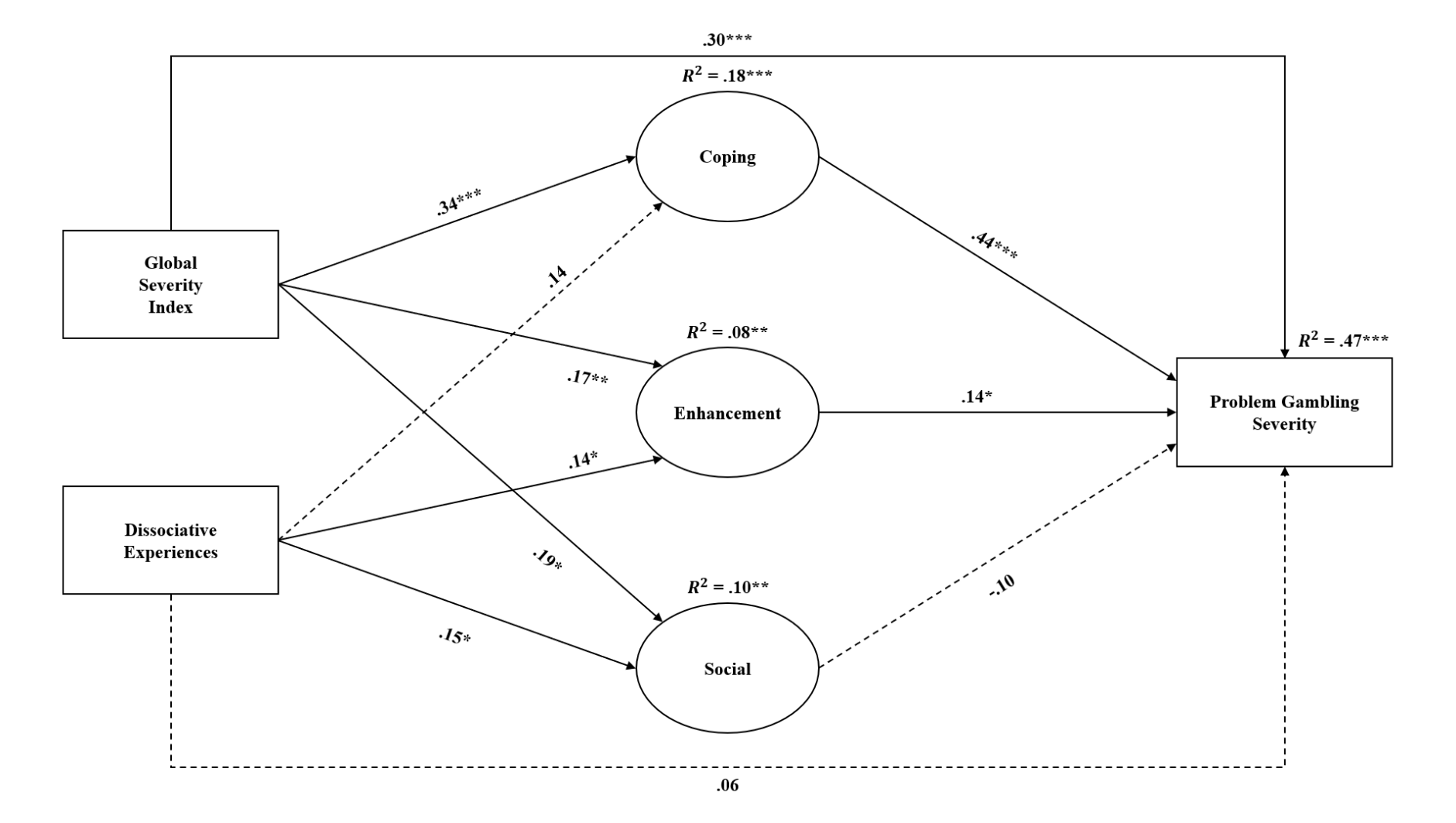
The overall mediation model (males) with standardized path coefficients and the explained variance of the endogenous variables (R^2^) (N = 388).*

*Notes.* ML estimation with 10,000 bias-corrected bootstrap resamples was applied. All three mediator variables are latent variables. Also, for clarity, the covariances between the errors of all mediator variables have not been depicted in the figure. Simple arrows: significant path coefficients, dotted arrows: nonsignificant path coefficients. **p*<.05; ***p*<.01; ****p*<.001

**Figure S2**

*
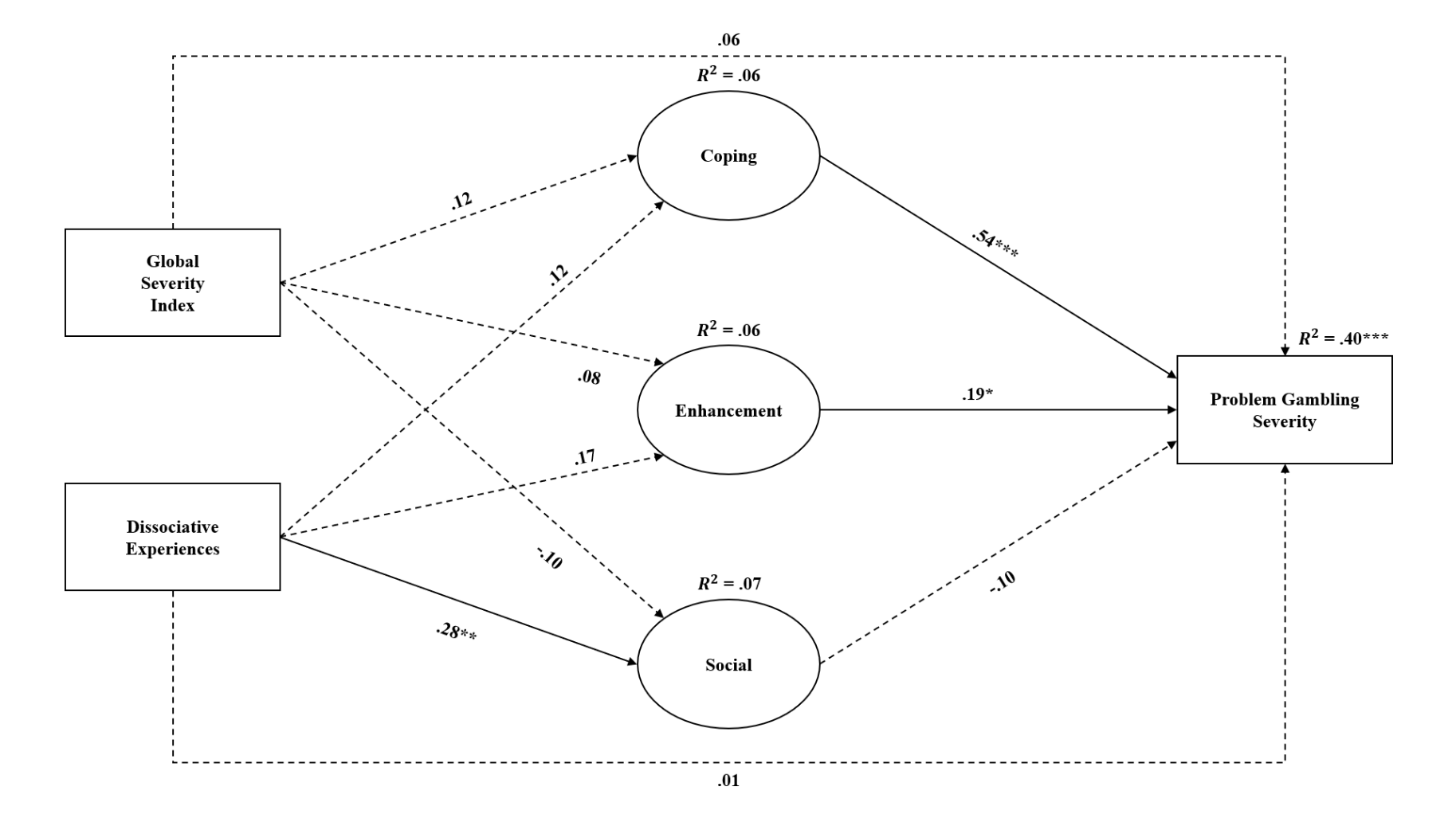
The overall mediation model (females) with standardized path coefficients and the explained variance of the endogenous variables (R^2^) (N = 279).*

*Notes.* ML estimation with 10,000 bias-corrected bootstrap resamples was applied. All three mediator variables are latent variables. Also, for clarity, the covariances between the errors of all mediator variables have not been depicted in the figure. Simple arrows: significant path coefficients, dotted arrows: nonsignificant path coefficients. **p*<.05; ***p*<.01; ****p*<.001
